# “Classical cytogenetics” is not equal to “banding cytogenetics”

**DOI:** 10.1186/s13039-017-0305-9

**Published:** 2017-02-16

**Authors:** Thomas Liehr

**Affiliations:** Institute of Human Genetics, Jena University Hospital, Friedrich Schiller University, Kollegiengasse 10, D-07743 Jena, Germany

**Keywords:** Classical cytogenetics, Banding cytogenetics, Research, Diagnostics, ISCN

## Abstract

**Background:**

Human cytogenetics is a field suffering from the argumentation that it ‘is nowadays really outdated and to be replaced by molecular high throughput approaches’. Thus, it is to be expected that non-cytogeneticists do mistakes in nomenclature of cytogenetics, which is exposed to repeated reforms, like e.g. recently the now hardly manageable and readable nomenclature for array-comparative genomic hybridization.

**Results:**

An unexpected nomenclature problem becomes more and more obvious in human cytogenetics – it seems to become difficult to understand how and when to use the designations “classical cytogenetics” or “banding cytogenetics”. Here it is highlighted that “classical cytogenetics” stands for studies undertaken by Orcein or Giemsa staining without (!) previous trypsin-treatment. However, in human (diagnostic) cytogenetics almost exclusively “banding cytogenetics” is applied.

**Conclusion:**

The terms “classical cytogenetics” and “banding cytogenetics” have to be clearly distinguished and correctly applied.

As Editor of Molecular Cytogenetics I felt that it may be necessary to provide to the community of our field the following commentary:

Cytogenetics is a field now being ~130 year old [1]. Thus, we should expect it to be a well-accepted and established area, where everyone working in genetics is aware of its advantages and unique possibilities (see Table 1). Well, we all know that this is not the case, unfortunately, as our field suffers from the argumentation that it is outdated and to be replaced by more modern approaches soon [2–7]. This was even already the case when I entered this field in 1991, and already before, when my supervisor Prof. Erich Gebhart (Erlangen, Germany) published his first articles in 1968 [8], as he told me. In Germany there is a saying: “People declared dead live longer”; so this seems to be valid for (molecular) cytogenetics, too. For example, it is now realized that the karyotype codes “system inheritance” or the overall blueprint, which serves as an important tool to monitor genome instability in cancer and other diseases/illnesses. Moreover, since chromosomes and genes represent different levels of genetic information, karyotype analysis should not be replaced by DNA sequencing, as the more exciting phase of molecular cytogenetics is yet to come [7].

**Table 1:** Advantages and unique features of (molecular) cytogenetics compared to other approaches

Features of (molecular) cytogenetics
1. Analyses on single cell level
2. Analyses of whole genome
3. Quick and simple detection of gross genetic alterations
4. All kinds of structural changes can be detected irrespective if balanced or unbalanced
5. Analyses ‘in situ’ not ‘in vitro’
6. Sex mismatched contamination can be detected easily
7. Low level mosaics are detectable on routine bases
8. Monitor cell population stability and heterogeneity by comparing the rates of clonal and non clonal karyotypes

It is not at all surprising, that non-specialists for our field do mistakes in nomenclature of cytogenetics, which is based on the “international system for human cytogenetic nomenclature” (ISCN), or how it was recently renamed “international system for human cytogenomic nomenclature” (also ISCN!?) [9]. ISCN was and still is in parts difficult to understand and matter of discussion [10–13]. Also some recent reforms of e.g. the array comparative genomic hybridization nomenclature led to profound problems with readability, as important hyphens were abandoned; two examples are given in Table 2.

**Table 2:** Comparison of readability of microarray-based chromosome nomenclature of 2013 [17] and 2016 edition of ISCN [9] in two examples

	Nomenclature acc. to 2013 edition of ISCN [17]	Nomenclature acc. to 2016 edition of ISCN [9]
Example 1	arr[hg19] 12p13.33p11.1(84,917-34,382,567)x3	arr[GRCh37] 12p13.33p11.1(84917_34382567)x3
Example 2	arr[hg19] 21q11.2q22.3(9,931,865-46,914,745)x1	arr[GRCh37] 21q11.2q22.3(9931865_46914745)x1

However, it is really awkward to see that even people studying chromosomes with devotion and publish in parts extremely interesting results, seem not to be aware any more of a simple nomenclature based on the history of cytogenetics; i.e. when to use the designations “classical cytogenetics” and “banding cytogenetics”. Already in 2002 I summarized: “History of human cytogenetics can be divided into three major time periods: the pre-banding era (1879–1970), the pure banding era (1970–1986) and the molecular cytogenetic era (1986–today). The prebanding era is characterized by the first visualization the word “chromosome” (from chroma = color and soma = body) in 1888, the determination of the correct modal human chromosome number in 1956 and the detection of the first chromosomal abnormality in Down syndrome in 1959. The banding era started with the discovery of the Qbanding method by Dr. Lore Zech (Upsala) in 1970 [14]. Many more chromosomal abnormalities, such as translocations, inversions, deletions and insertions, could be detected from now on. Currently, the GTG-banding approach (G-bands by Trypsin using Giemsa) [15] is still the gold-standard for all cytogenetic techniques. However, the pure banding era ended in 1986 with the first molecular cytogenetic experiment on human chromosomes” [1].

For the nomenclature problem raised here it is important to recall that the pre-banding era was characterized by the exclusive ability to stain human chromosomes in one color, e.g. Orcein or Giemsa staining without (!) trypsin-treatment. In case someone does a study like that, which is still routinely done in many animal chromosomes [16], or in mutagenesis studies [8], he performs a “classical cytogenetic study”. Still, nowadays no-one will do “classical cytogenetics” in human clinical diagnostic applications, as here we routinely apply “banding cytogenetics”! Unfortunately, it is not hard to find published studies where this difference was not considered (I intentionally do not refer to them here). Also as editor and referee I get more and more submissions with the statement ‘we did classical cytogenetics in this clinical case’. And I have to say then: ‘no you did not, you did banding cytogenetics; please correct that point before we can accept your publication’.

So I herewith want to appeal to all specialists doing banding cytogenetics and who publish or talk about it, please denominate correctly the approach you use. Remember and teach to your students that “classical cytogenetics” is not equal to “banding cytogenetics”.

Thanks a lot.

## Abbreviations

GTG: G-bands by Trypsin using Giemsa
